# Long non-coding RNA MEG3 acts as a suppressor in breast cancer by regulating miR-330/CNN1

**DOI:** 10.18632/aging.205419

**Published:** 2024-01-17

**Authors:** Dandan Yi, Zetian Wang, Haojie Yang, Ru Wang, Xianbiao Shi, Zhijian Liu, Fazhan Xu, Qing Lu, Xiao Chu, Jianfeng Sang

**Affiliations:** 1 Department of General Surgery, Nanjing Drum Tower Hospital, Affiliated Hospital of Medical School, Nanjing University, Nanjing 210008, China; 2 Department of Trauma-Emergency and Critical Care Medicine, Shanghai Fifth People’s Hospital, Fudan University, Shanghai 200240, China; 3 Department of Coloproctology, Yueyang Hospital of Integrated Traditional Chinese and Western Medicine, Shanghai University of Traditional Chinese Medicine, Shanghai 200437, China; 4 Department of General Surgery, Nanjing Drum Tower Hospital, Clinical College of Nanjing Medical University, Nanjing 210008, China; 5 Department of Breast Surgery, Yueyang Hospital of Integrated Traditional Chinese and Western Medicine, Shanghai University of Traditional Chinese Medicine, Shanghai 200437, China; 6 Department of Thoracic Surgery, The Fifth People’s Hospital of Shanghai, Fudan University, Shanghai 200240, China

**Keywords:** MEG3, breast cancer, miR-330, CNN1, calponin

## Abstract

Background: The current study aimed to investigate the molecular mechanism of long non-coding RNA (lncRNA) MEG3 in the development of breast cancer.

Methods: The regulating relationships among lncRNA MEG3, miRNA-330 and CNN1 were predicted by bioinformatics analysis of breast cancer samples in the Cancer Genome Atlas database. The differential expression of lncRNA MEG3, miRNA-330 and CNN1 was first validated in breast cancer tissues and cells. The effects of lncRNA MEG3 on breast cancer malignant properties were evaluated by manipulating its expression in MCF-7 and BT-474 cells. Rescue experiments, dual-luciferase assays, and RNA immunoprecipitation (RIP) experiments were further used to validate the relationships among lncRNA MEG3, miRNA-330 and CNN1.

Results: Bioinformatics analysis showed that lncRNA MEGs and CNN1 were significantly downregulated in breast cancer tissues, while miR-330 was upregulated. These differential expressions were further validated in our cohort of breast cancer samples. High expression levels of lncRNA MEG3 and CNN1 as well as low expression of miR-330 were significantly associated with favorable overall survival. Overexpression of lncRNA MEG3 significantly inhibited cell viability, migration and invasion, decreased cells in S stage and promoted cell apoptosis. Dual-luciferase reporter gene assay and RIP experiments showed that lncRNA MEG3 could directly bind to miR-330. Moreover, miR-330 mimics on the basis of lncRNA MEG3 overexpression ameliorated the tumor-suppressing effects of lncRNA MEG3 in breast cancer malignant properties by decreasing CNN1 expression.

Conclusion: Our study indicated lncRNA MEG3 is a breast cancer suppressor by regulating miR-330/CNN1 axis.

## INTRODUCTION

Breast cancer is the most common cancer that threatens female’s health all over the world. Its global incidence surpassed lung cancer as the first in female malignancy, accounting for over 2.3 million new cases in 2020 [1]. Breast cancer contributes significantly to the cancer-related death in female [2], especially the most malignant subtype, triple negative breast cancer. Currently, treatment strategies for breast cancer include surgery, chemotherapy, radiation, and endocrine therapy [3]. However, the effect is still not satisfied due to metastasis, recurrence and drug resistance [4]. Especially, approximately 60% breast cancer are diagnosed at advanced stage, resulting a high chance of mortality [5]. Therefore, investigating the underlying genetic mechanisms to identify novel candidate therapeutic targets is urgent for improving patient outcomes.

Competing endogenous RNAs (ceRNAs) refers to a class of non-coding RNAs, including circular RNAs and long non-coding RNAs (lncRNAs) that can bind to the target mRNAs by competing with microRNAs (miRNAs) to regulate the transcriptional level of mRNAs [6]. Considerable researches have been made in recent years and found several circular RNAs or lncRNAs that play pivotal roles in various disease progression [7, 8]. For example, lncRNA DLEU2 acts as a miR-181a sponge to regulate SEPP1 and inhibit skeletal muscle differentiation and regeneration [9]. LncRNA GPC5-AS1 stabilizes GPC5 mRNA by competitively binding with miR-93/106a to suppress gastric cancer [10]. Circ_0000423 regulates synthesis of cartilage extracellular matrix by miRNA-27b-3p/MMP-13 axis in osteoarthritis [11]. Circ_RPPH1 hinders the growth and metastasis of breast carcinoma cells by mediating miR-16b-3p/E2F2 pathway [12]. Circ RNA_0006014 promotes breast cancer progression by sponging miR-885-3p to regulate NTRK2 and PIK3/AKT pathway [13]. In recent years, ceRNA networks have become a novel research focus and have facilitated the identification of potential prognostic biomarkers and therapeutic targets owing to the development of transcriptomics, bioinformatics and big data science [14–16]. A recent study investigated differential lncRNAs and ceRNA networks in triple-negative breast cancer and identified three lncRNAs as prognostic biomarkers [17]. Moreover, comprehensive transcriptomic analysis was performed to decipher ceRNA regulatory networks in endocrine-resistant breast cancer [18]. Additionally, the circular RNA-miRNA-mRNA networks involved in the tumorigenesis of breast cancer have been explored [19]. However, the molecular targets identified by bioinformatics analyses in these studies often lack validation experiments, and their functions in the biology of breast cancer should be further explored.

Here, we performed comprehensive bioinformatics analyses on RNA sequencing data and miRNA sequencing data of breast cancer to establish a ceRNA network. Further, the regulatory relationships among these genes were validated and their effects on breast cell proliferation, apoptosis, migration, and invasion were also investigated.

## RESULTS

### ceRNA network construction

After data annotation, we obtained 17141 mRNAs, 187 lncRNAs, and 1488 miRNAs from raw data downloaded from the UCSC Xena database. We identified 3556 differentially expressed mRNAs (DEmRNAs) (2549 upregulated and 1007 downregulated mRNAs, Supplementary Figure 1A), 105 differential lncRNAs (73 upregulated and 32 downregulated lncRNAs, Supplementary Figure 1C), and 485 differential miRNAs (196 upregulated and 289 downregulated miRNAs, Supplementary Figure 1E) between tumor and normal samples. Heatmaps of the top 10 DEmRNAs, differential lncRNAs, and differential miRNAs are shown in Supplementary Figure 1B, 1D, and 1F, respectively. The complete list of differentially expressed genes are displayed in Supplementary Table 1.

These identified DEmRNAs were significantly enriched in several Gene Ontology (GO)- biological process terms associated with “cell adhesion”, “biological adhesion” and “cell-cell signalling” (Supplementary Figure 2). The upregulated genes were significantly enriched in 55 signaling pathways, including “PI3K-Akt signaling pathway”, “cAMP signaling pathway”, “RAP1 signaling pathway”, whereas the downregulated genes were significantly enriched in six signaling pathways, such as “Proteasome” and “DNA replication” (Supplementary Figure 3).

**Figure 1:**
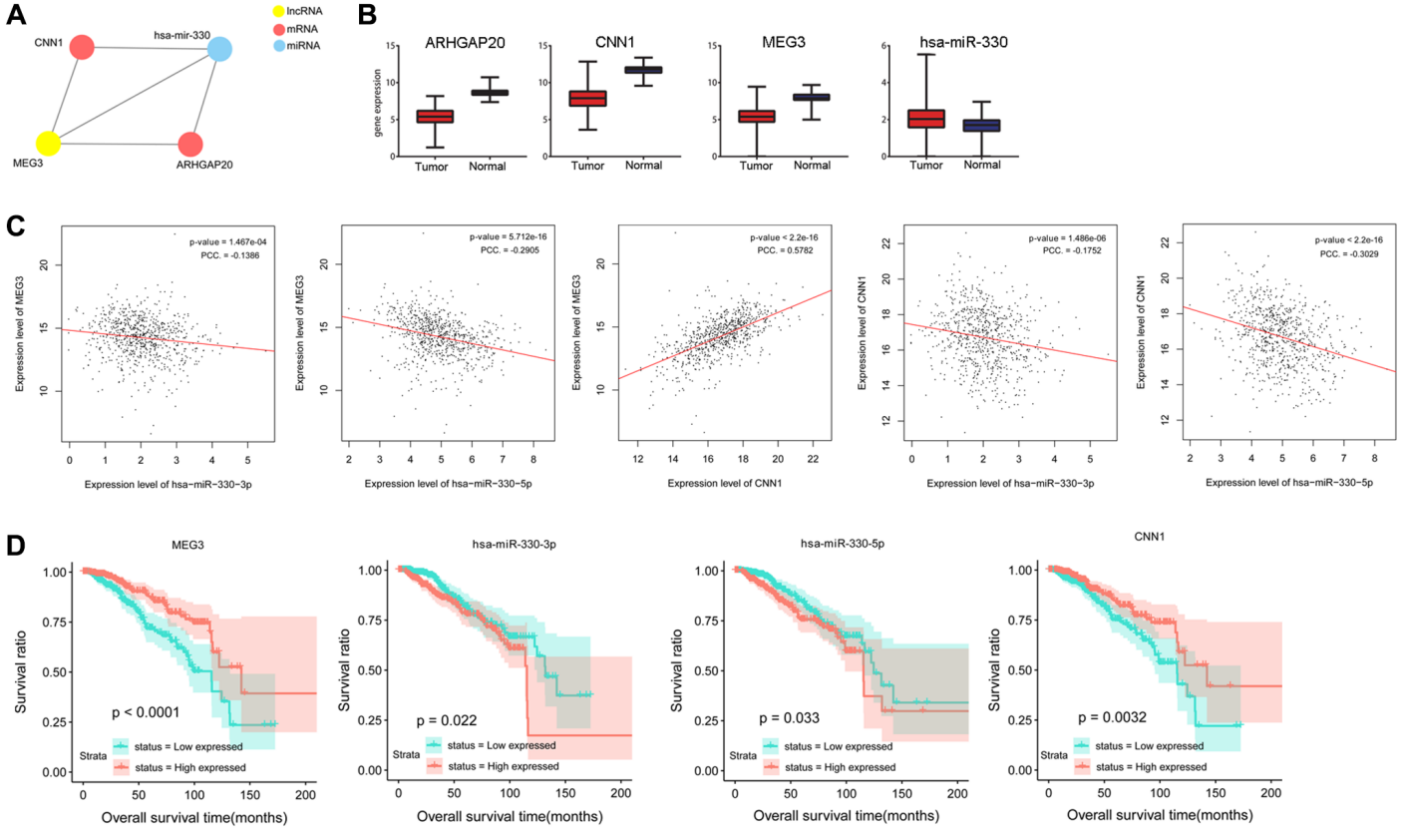
**Prediction and validation of a ceRNA network consisting miR-330, lncRNA MEG3, and CNN1.** (**A**) A ceRNA network composed of lncRNA MEG3, miR-330, CNN1 and ARHGAP20. (**B**) Gene expression of lncRNA MEG3, miR-330, CNN1 and ARHGAP20 of tumor and normal samples in TCGA. (**C**) Correlation analysis was performed between expression levels of lncRNA MEG3, miR-330, and CNN1. (**D**) Overall survival time was significantly different between high expression samples and low expression samples of lncRNA MEG3, miR-330, and CNN1. Abbreviation: PCC: Pearson correlation coefficient.

By integrating the relationships among differential lncRNAs, differential miRNAs and DEmRNAs, a ceRNA network was constructed, including CNN1, ARHGAP20, MEG3, and miR-330 (Figure 1A). CNN1 (log2FC = −3.885), ARHGAP20 (log2FC = −3.312), and MEG3 (log2FC = −2.612) were downregulated, whereas miR-330 (log2FC = 0.431) was upregulated in tumor samples compared to normal samples (Figure 1B). Figure 1C shows that MEG3 expression was negatively correlated with miR-330-3p expression (*P* = 1.467e-04, PCC = −0.1386) and miR-330-5p expression (*P* = 5.712e-16, PCC = −0.2905), but was positively correlated with CNN1 expression (*P* < 2.2e-16, PCC = 0.5782). Additionally, miR-330-3p expression (*P* = 1.486e-06, PCC = −0.1752) and miR-330-5p expression (*P* < 2.2e-16, PCC = −0.3029) were negatively correlated with CNN1 expression. Based on these results, we speculated that MEG3 might act as a ceRNA to regulate CNN1 and ARHGAP20 via sponging miR-330.

Survival analysis showed that miR-330-3p and miR-330-5p expression levels were negatively associated with the overall survival of patients (*P* = 0.022, *P* = 0.033), whereas MEG3 and CNN1 expression levels were positively associated with survival time of patients (*P* < 0.0001, *P* = 0.0032, Figure 1D).

### lncRNA MEG3 and CNN1 were upregulated in breast cancer tissues and cells, while miR-330 was downregulated

**Figure 2:**
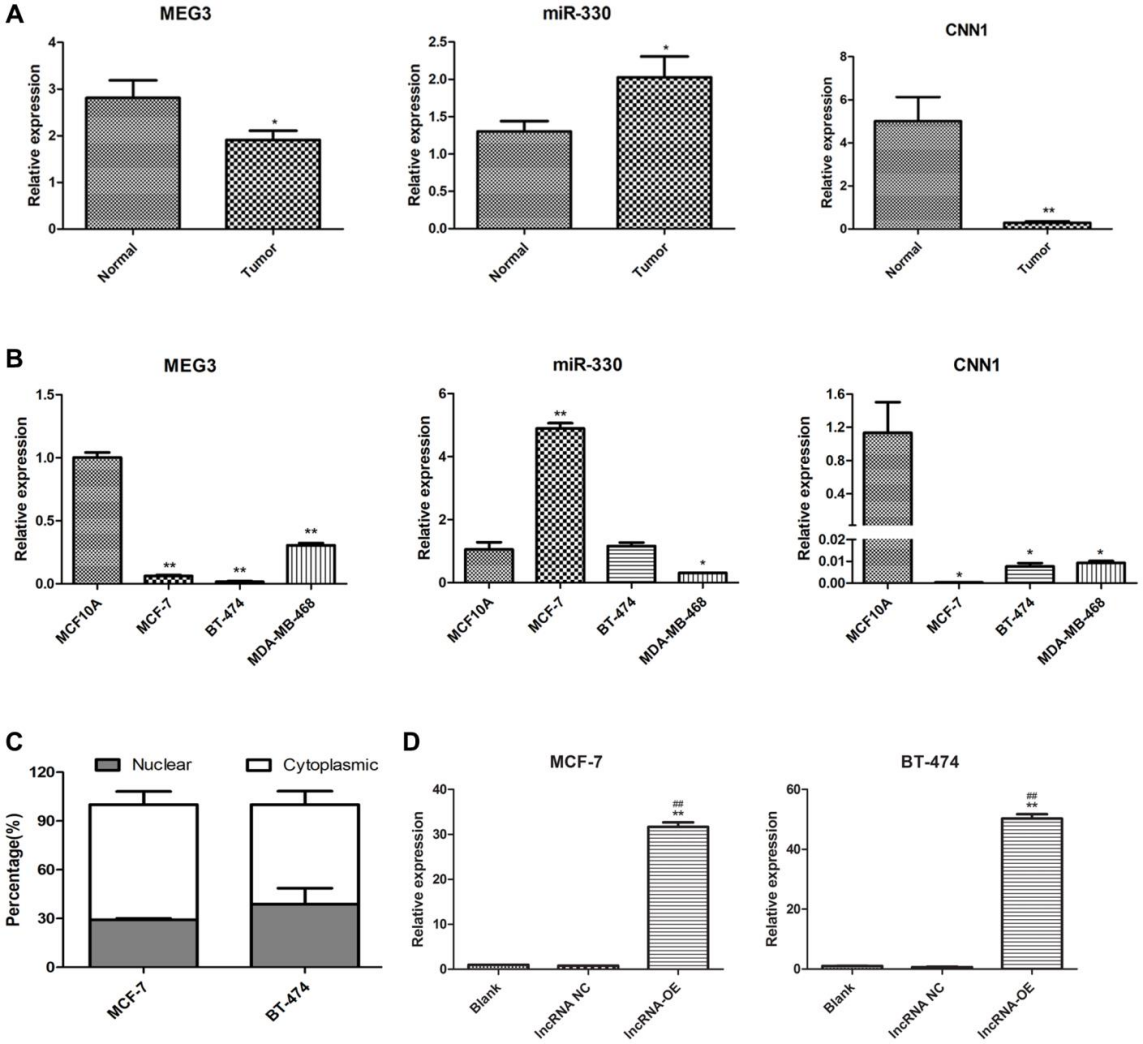
**Expression of lncRNA MEG3, and CNN1 in breast cancer tissues and cells.** (**A**) qRT-PCR detected the expression of lncRNA MEG3, miR-330, and CNN1 in tumor and normal tissue of our cohort. ^*^P < 0.05, ^**^P < 0.01 vs. para-tumor tissue (Normal). (**B**) mRNA expression of miR-330, lncRNA MEG3, and CNN1 in MCF10A, MCF-7, BT-474, and MDA-MB-468 cells. ^*^P < 0.05, ^**^P < 0.01 vs. MCF10A cells. (**C**) Subcellular location of lncRNA MEG3 in MCF-7 and BT-474 cells. (**D**) lncRNA MEG3 was overexpressed in MCF-7 and BT-474 cells. lncRNA MEG3 expression was detected by qRT-PCR. ^**^P < 0.01 vs. Blank, ^##^*P* < 0.01 vs. lncRNA NC. Abbreviations: NC: negative control; OE: overexpression.

The qPCR analysis was performed to examine the expression levels of MEG3, miR-330, and CNN1 in tumor tissues and cells. Results showed that miR-330 was significantly upregulated (*P* < 0.05), whereas MEG3 (*P* < 0.05) and CNN1 (*P* < 0.01) were significantly downregulated in tumor tissues compared to paratumor tissue at the RNA level (Figure 2A). Cell experiments showed consistent results that MEG3 (*P* < 0.01) and CNN1 expression levels (*P* < 0.05) were markedly decreased in human breast cancer cells MCF-7, BT-474, and MDA-MB-468 compared to those in non-cancerous human breast epithelial cells MCF10A. However, an obvious elevation in miR-330 was observed in MCF-7 cells only (*P* < 0.01; Figure 2B). Taken together, the results of tumor tissue and cell experiments were consistent with the results of the bioinformatics analyses. Moreover, nuclear-cytoplasmic fractionation revealed that MEG3 was primarily present in the cytoplasmic compartment (Figure 2C). Because MEG3 was markedly downregulated in MCF-7 and BT-474 cells, these cells were transfected with lncRNA MEG3-overexpressing plasmid. qPCR validated the successful overexpression of MEG3 MCF-7 cells and BT-474 cells, in comparison with the blank control or negative control (*P* < 0.01, Figure 2D).

### lncRNA MEG3 overexpression inhibited malignant properties of breast cancer

**Figure 3:**
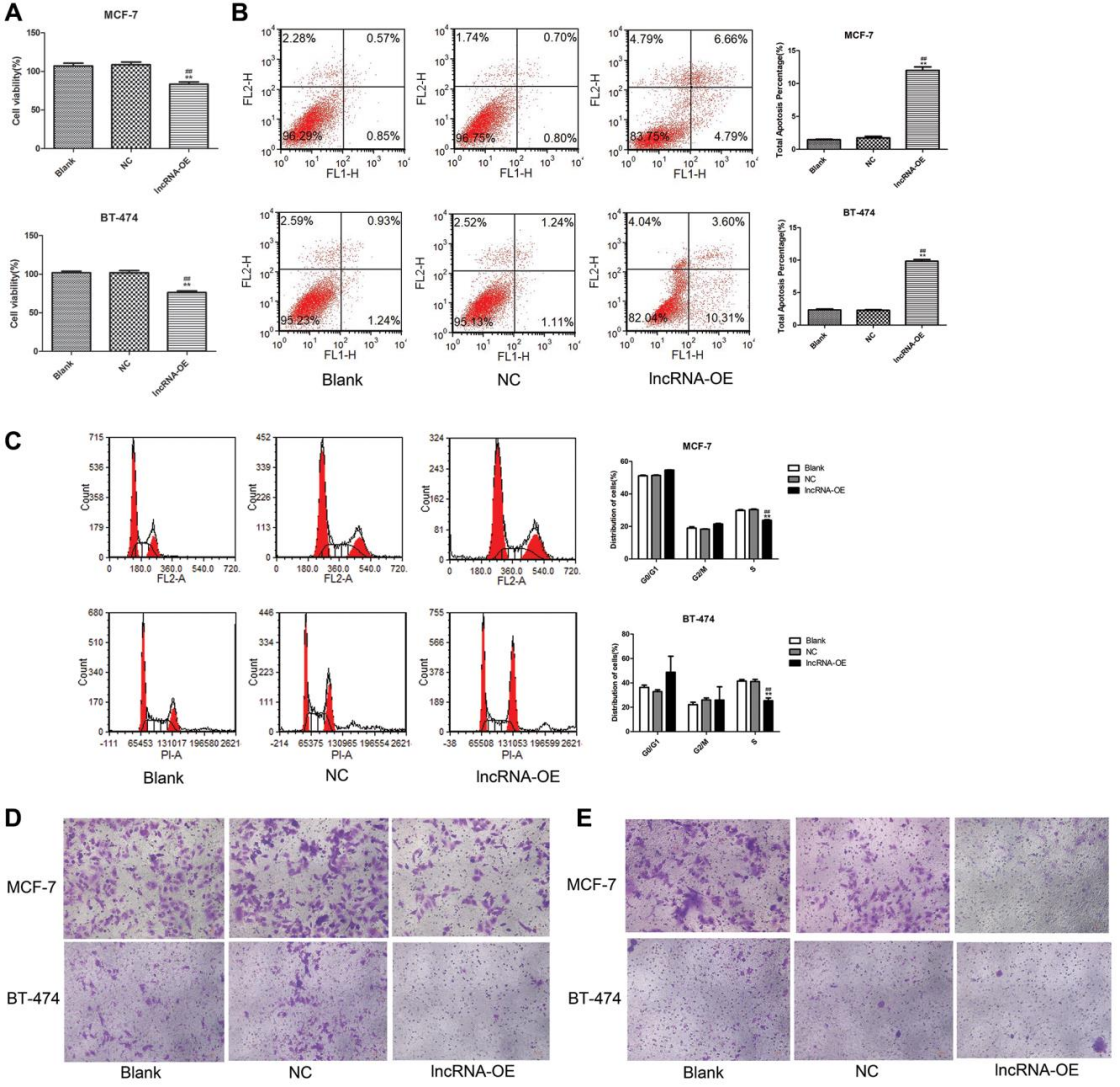
**Overexpression of lncRNA MEG3 on cell viability, apoptosis, migration and invasion.** (**A**) CCK-8 assay evaluated the effect of lncRNA MEG3 overexpression on cell viability. (**B**) Flow cytometry evaluated the effect of lncRNA MEG3 overexpression on cell apoptosis. (**C**) Flow cytometry evaluated the effect of lncRNA MEG3 overexpression on cell cycle. (**D**) Transwell detected the effect of lncRNA MEG3 overexpression on cell migration (magnification, 200×). (**E**) Transwell detected the effect of lncRNA MEG3 overexpression on cell invasion (magnification, 200×); ^**^P < 0.01 vs. Blank; ^##^*P* < 0.01 vs. lncRNA NC.

We found that MEG3 overexpression significantly decreased cell viability (*P* < 0.01, Figure 3A), increased cell apoptosis (*P* < 0.01, Figure 3B), decreased the number of cells at S stage (*P* < 0.01, Figure 3C), and suppressed cell migration (Figure 3D) and cell invasion (Figure 3E) in both MCF-7 and BT-474 cells.

### lncRNA MEG3 regulated CNN1 expression by sponging miR-330

**Figure 4:**
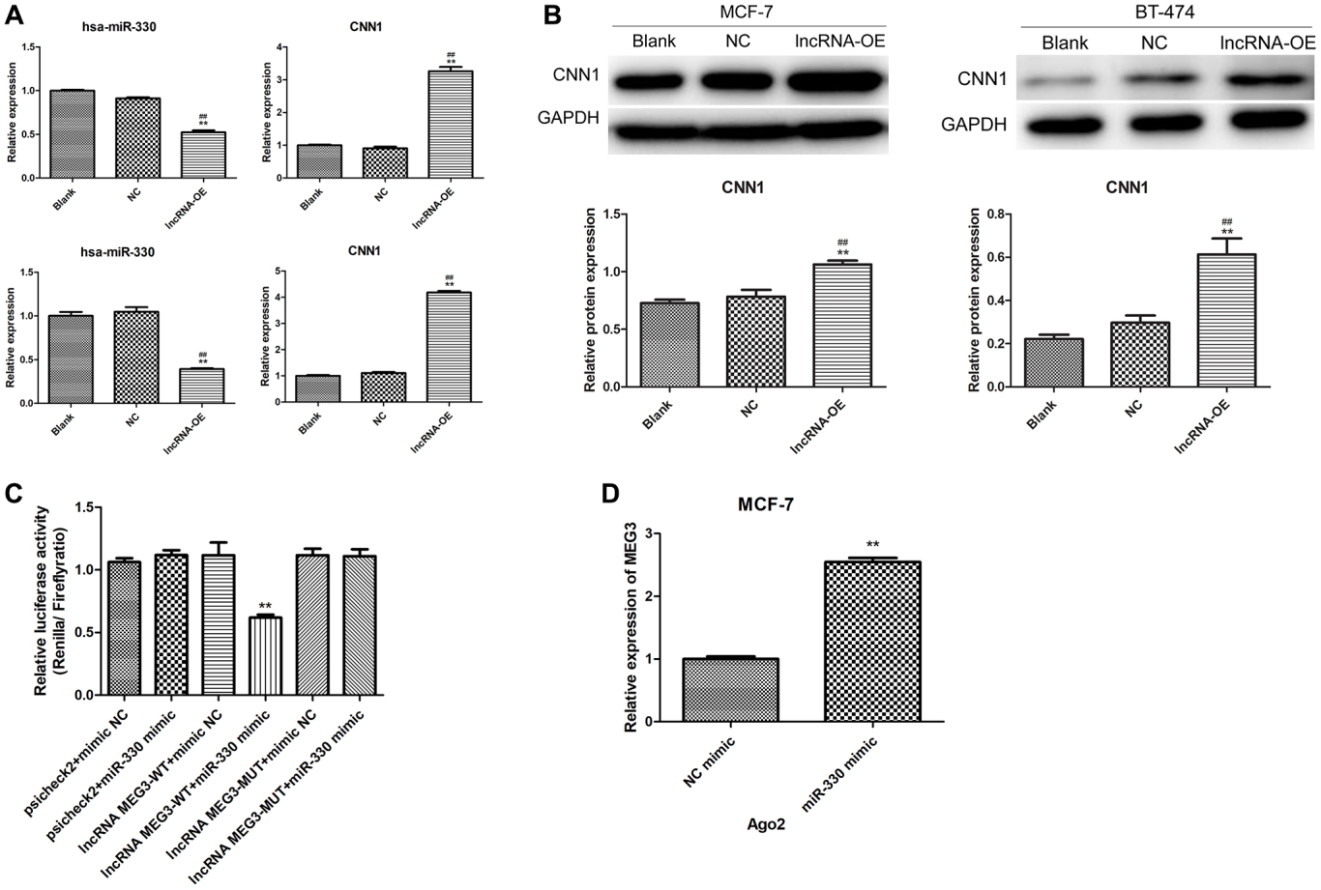
**Validation of interaction among lncRNA MEG3, miR-330 and CNN1.** (**A**) Effect of lncRNA MEG3 overexpression on miR-330 expression and CNN1 mRNA expression. Up panel, MCF-7 cells; below panel, BT-474 cells. (**B**) Effect of lncRNA MEG3 overexpression on CNN1 protein expression. ^**^P < 0.01 vs. blank; ^##^*P* < 0.01 vs. lncRNA NC. (**C**) Luciferase reporter assay. ^**^P < 0.01 vs. psicheck2 + mimic NC. (**D**) lncRNA MEG3 binds to miR-330 by RIP assay. ^**^P < 0.01 vs. NC mimic.

The effect of MEG3 overexpression on miR-330 and CNN1 expression was further investigated in MCF-7 and BT-474 cells. Overexpression of MEG3 led to a significant increase in CNN1 expression and a remarkable decrease in miR-330 expression in both MCF-7 cells and BT-474 cells (*P* < 0.01, Figure 4A). Similarly, MEG3 overexpression markedly increased CNN1 protein expression in both MCF-7 and BT-474 cells (*P* < 0.01, Figure 4B). These findings indicated that MEG3 positively regulated CNN1 expression and negatively regulated miR-330 expression in breast cancer cells.

Dual-luciferase reporter gene assays and RIP experiments were performed to investigate the relationship between lncRNA MEG3 and miR-330. The dual-luciferase reporter gene assay confirmed the targeting correlation between MEG3 and miR-330 (Figure 4C). The RIP experiments showed that MEG3 interacts with Ago2, an RNA binding protein, and acts as a sponge for miR-330 (Figure 4D).

### miR-330 mimics counteracted the effects of lncRNA MEG3 overexpression on CNN1 expression and malignant properties of breast cancer

**Figure 5:**
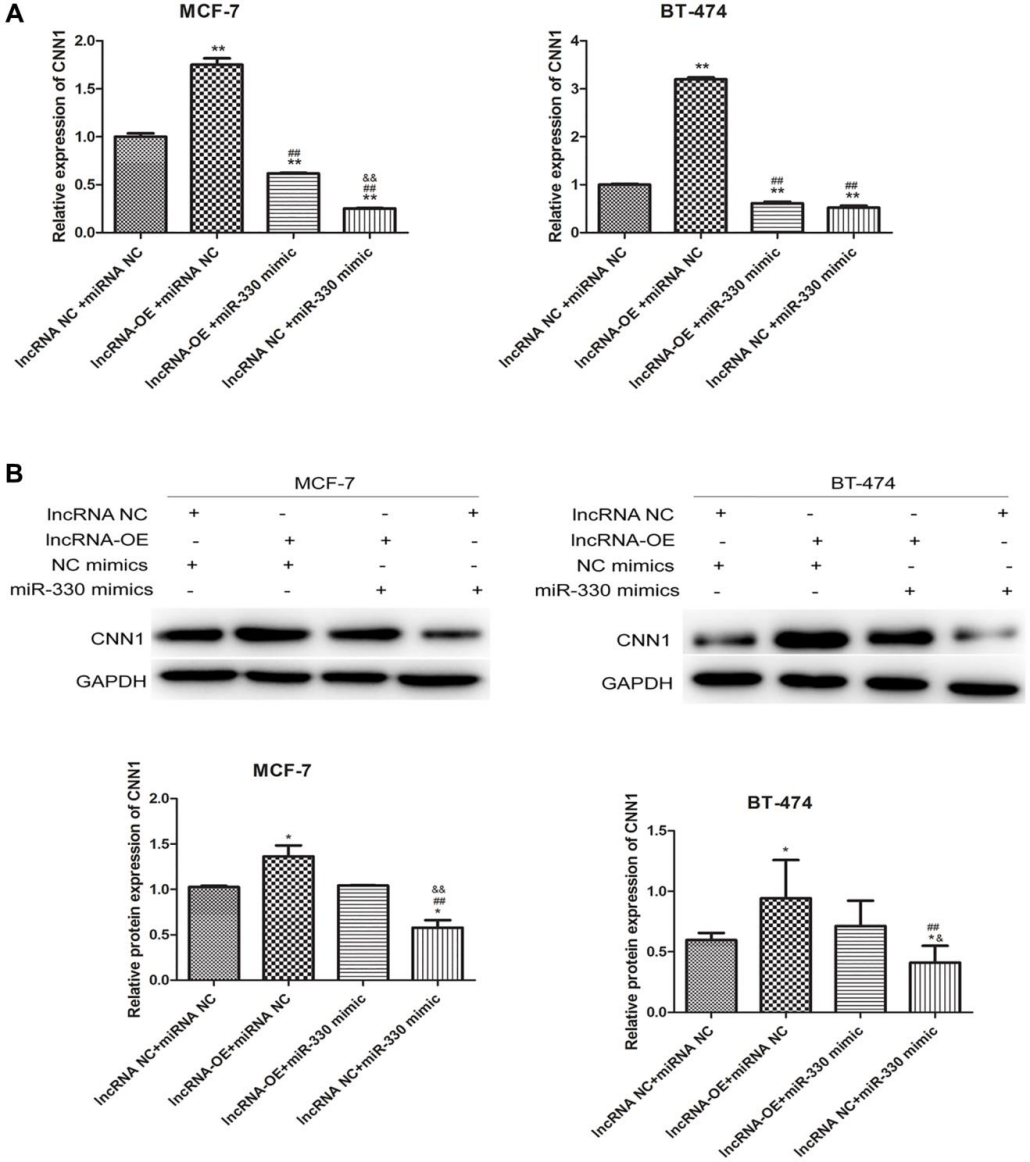
lncRNA MEG3 overexpressing-induced CNN1 up-regulation at mRNA level (**A**) and protein level (**B**) was abolished by miR-330 mimics.

To further elucidate the relationship between MEG3, miR-330 and CNN1, we treated the lncRNA MEG3-overexpressing cells with miR-330 mimics to upregulate miR-330. miR-330 mimics could partly abolish the significant increases in mRNA (*P* < 0.01, Figure 5A) and protein expression of CNN1 induced by MEG3 overexpression (*P* < 0.05, Figure 5B).

**Figure 6:**
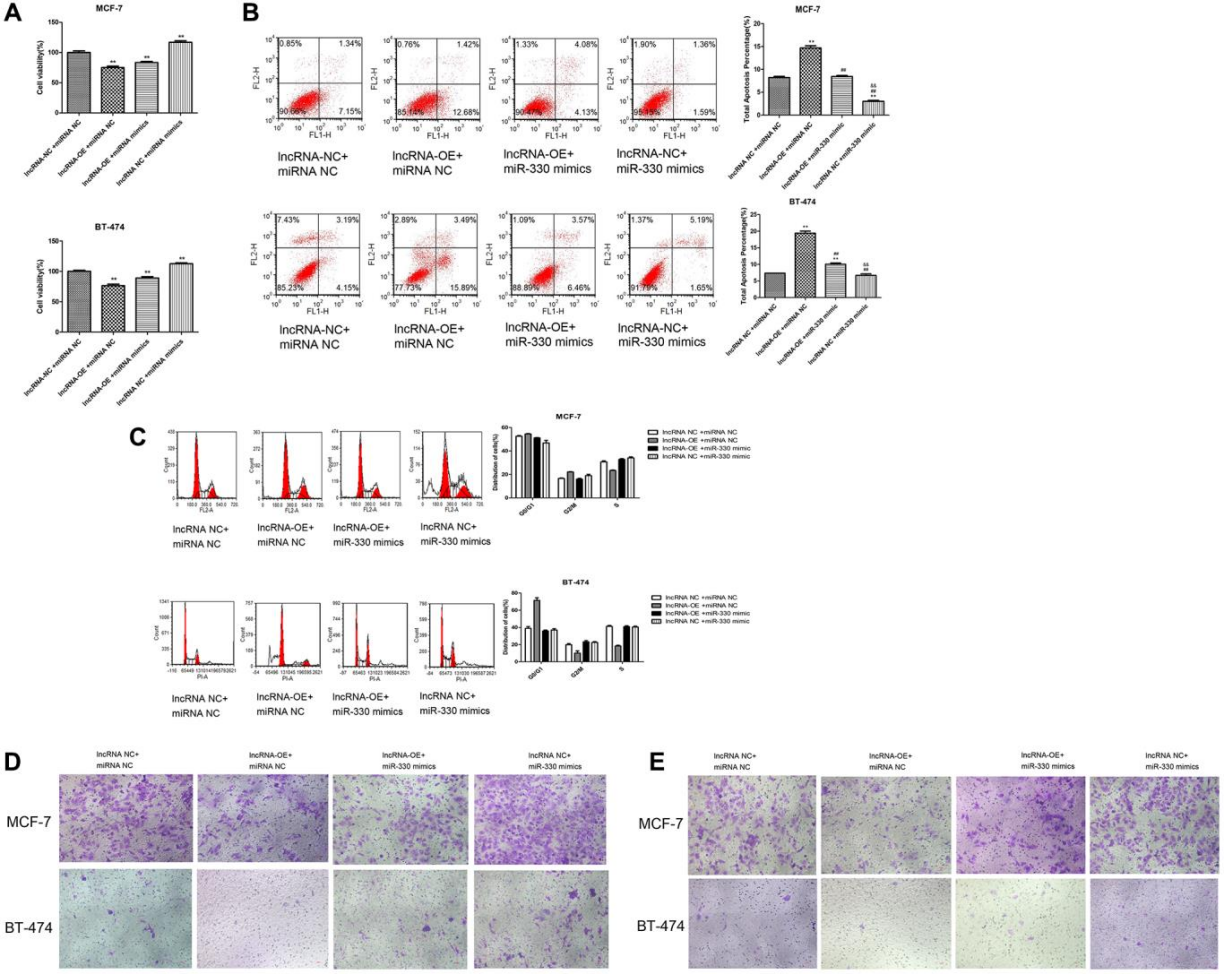
**Overexpression of lncRNA MEG3 on cell viability, apoptosis, migration and invasion by regulating miR-330/CNN1.** (**A**) Decrease in cell viability caused by lncRNA MEG3 overexpression was partly counteracted by miR-330 mimics. ^*^P < 0.05, ^**^P < 0.01 vs. lncRNA NC + miRNA NC; ^##^*P* < 0.01 vs. lncRNA-OE+ miRNA NC; ^&&^*P* < 0.01 vs. lncRNA-OE + miRNA NC. (**B**) Effect of co-treatment of lncRNA MEG3 overexpression and miR-330 mimics on cell apoptosis. (**C**) Effect of co-treatment of lncRNA MEG3 overexpression and miR-330 mimics on cell cycle. ^**^P < 0.01 vs. lncRNA NC + miRNA NC; ^##^*P* < 0.01 vs. lncRNA-OE + miRNA NC; ^&&^*P* < 0.01 vs. lncRNA-OE + miRNA NC. (**D**) Effect of co-treatment of lncRNA MEG3 overexpression and miR-330 mimics on cell migration. (**E**) Effect of co-treatment of lncRNA MEG3 overexpression and miR-330 mimics on cell invasion (magnification, 200×).

Moreover, we found that miR-330 mimics increased cell viability (*P* < 0.01, Figure 6A), decreased the number of apoptotic cells (*P* < 0.01, Figure 6B), increased the cells at S stage (*P* < 0.05, Figure 6C), and promoted cell migration (Figure 6D) and cell invasion (Figure 6E) in lncRNA MEG3-overexpressing MCF-7 and BT-474 cells.

## DISCUSSION

Previous studies have documented that ceRNAs are involved in regulating the expression of oncogenes and tumour suppressor genes at the post-transcriptional level through a miRNA-mediated mechanism, thus contributing to cancer [20, 21]. In the present study, we performed an integrated analysis of ceRNA networks in breast cancer by performing comprehensive bioinformatics analysis, tumour tissue analysis, and cell experiments. By analyzing the gene and miRNA expression profiles of breast cancer from the Cancer Genome Atlas database, 3556 DEmRNAs, 105 differential lncRNAs, and 485 differential miRNAs were identified between tumour and normal samples. We constructed a ceRNA network comprising lncRNA MEG3, miR-330, and CNN1, implying that MEG3 plays a ceRNA role in regulating CNN1 by sponging miR-330 in breast cancer. *In vitro* experiments verified these relationships and suggested that MEG3 suppresses cell proliferation, migration, and invasion, and promotes cell apoptosis via miR-330/CNN1 axis. The present study helps to deepen our understanding of the regulatory roles and biological behaviours of ceRNAs in the pathogenesis of breast cancer and reveals promising diagnostic and therapeutic targets.

It has been established that lncRNAs may function as oncogenes or tumour suppressors in the progression and metastasis of breast cancer and have great potential as diagnostic and prognostic biomarkers [22]. An increasing number of studies have revealed that MEG3 is downregulated and represses cell proliferation, migration, and invasion, and induces apoptosis in breast cancer cells [23–27]. The present study confirmed the downregulation of MEG3 in both tumour tissue and breast cancer cells, showing consistent effects on cell proliferation, migration, invasion, and apoptosis. Previous studies have found that miR-330 is overexpressed in breast cancer and associated with poor prognosis [28–31], which is consistent with our finding. It has been reported that lncRNAs localized in the cytoplasm may act as sponges of some miRNAs to offset the effects of miRNAs [32]. Our study consistently found that MEG3 was predominantly distributed in the cytoplasm. Moreover, results of bioinformatics analysis and cell experiments indicated that MEG3 acts as a ceRNA of miR-330 and negatively regulates miR-330 expression. In addition, the RIP and dual-luciferase reporter assays demonstrated that MEG3 binds to miR-330. These results collectively suggest that MEG3 binds to miRNA-330 to interfere with its regulatory action, which is in accordance with a previous study by Shen et al., who reported that MEG3 sponges miR-330 in glioma [32].

The calponin family includes three isoforms: CNN1, CNN2, and CNN3. CNN1 is downregulated and has been shown to be a tumour suppressor gene in breast cancer, inhibiting cell proliferation and invasion, and enhancing cell apoptosis [33]. CNN1 downregulation in breast cancer cells was also observed in our study. The ceRNA network constructed in this study implied that CNN1 was targeted by the MEG3 and miRNA-330. Moreover, MEG3 overexpression led to CNN1 upregulation at the mRNA and protein levels, which was significantly reversed by treatment with miRNA-330 mimics. These results suggest that MEG3 plays a ceRNA role in regulating CNN1 by sponging miR-330 in breast cancer. Furthermore, our study showed that miRNA-330 mimics reduced the effects of MEG3 overexpression on cell proliferation, migration, invasion, and apoptosis. These results imply that the MEG3/miR-330/CNN1 axis may be a promising molecular mechanism involved in cell proliferation, migration, invasion, and apoptosis. Additionally, Wang et al. reported that CNN1 participates in mediating the effects of miR-106b-5p on metastasis of breast cancer via the Rho/ROCK1 signaling pathway [33]. It can be speculated that miR-106b-5p, Rho/ROCK1 signaling pathway, and AKT pathway may be important mediators involved in the lncRNA MEG3-related molecular mechanisms in breast cancer. However, further studies should be conducted to validate this speculation.

Several studies have shown that lncRNA MEG3 is a critical prognostic factor in patients with breast cancer [34, 35]. There is evidence that patients with high miR-330-3p expression have short survival times and miR-330-3p is an independent prognostic biomarker [31]. The prognostic value of CNN1 in breast cancer has also been reported [33]. However, the ceRNA relationship among lncRNA MEG3, miR-330 and CNN1 has not been reported. Consistent with these findings, our study found that MEG3 and CNN1 were positive prognostic factors, and miRNA-330 was a negative prognostic factor for breast cancer patients. These results validate the prognostic significance of MEG3, miRNA-330 and CNN1 in breast cancer and further broadening our understanding of their regulatory relationships.

There are some limitations in this study. First, animal experiments are still warranted to further certify the role of lncRNA MEG3-miR-330-CNN1 axis *in vivo*. Second, the downstream signaling pathways of CNN1 was not investigated in this study. Third, this study primarily focused on solid tissues, further experiments are needed to investigate the expression profiles of these genes in plasma to confirm whether they can be potential biomarkers for diagnosis and prognosis prediction of breast cancer.

In conclusion, our study demonstrated that lncRNA MEG3 acts as a sponge of miR-330 to target CNN1, thereby decreasing cell viability, increasing apoptotic cells, and suppressing cell migration and invasion. The MEG3/miR-330/CNN1 axis provides a novel insight into the pathogenesis of breast cancer and may represent candidate therapeutic targets.

## METHODS

### Establishing a ceRNA network using public data

Paired mRNA-seq data and miRNA-seq data, and the corresponding clinical characteristics of 468 breast cancer samples and 71 normal samples were obtained from TCGA database. Differentially expressed genes, including DEmRNAs, differential lncRNAs and differential miRNAs were filtered by the limma package (version 3.10.3) with Benjamini and Hochberg adjusted *P* < 0.05 and |log2fold change (FC)| >1. Further, GO enrichment analysis and Kyoto Encyclopedia of Genes and Genomes [36] pathway enrichment analysis were performed using the DAVID tool (version6.7) [37] and Gene Set Enrichment Analysis software (version 3.0), respectively (adjusted *P* < 0.05) [38].

Correlations between DEmRNAs and differential lncRNAs were analyzed by the corr.test function of the psych package in R with criteria of r ≥0.7 and adjusted *P* < 0.05. The relationship between differential lncRNAs and differential miRNAs were predicted by starBase (version 3.0) [39], and the relationships between differential miRNAs and DEmRNAs were predicted with the miRWalk online tool (version 3.0) [40]. Finally, these relationships were merged and screened the miRNA-mRNA pairs that were negatively regulated by the same DEmRNAs to construct a ceRNA network using Cytoscape software (version 3.7.0) [41]. The association between genes in the ceRNA network and overall survival were analyzed by Kaplan–Meier survival analysis with a log-rank *t*-test.

### Patients

Breast cancer tissues and adjacent normal tissues of 25 patients were collected at the Department of General Surgery of our hospital from 2019 to 2020. Diagnosis of the patients was confirmed by pathological biopsy. Enrolled patients were not treated with radiotherapy or chemotherapy within 3 months of enrollment. Ethical approval was obtained from the ethics committee of Nanjing Drum Tower Hospital, Clinical College of Nanjing Medical University. All experiments were performed in accordance with the Declaration of Helsinki. Written informed consent was obtained from all subjects.

### Cell culture and transfection

Breast cancer cells of MCF-7, BT-474, and MDA-MB-468 and normal breast cells MCF10A were cultured in DMEM/F12 (Gibco, batch number, 11995500BT) or RPMI1640 medium (Gibco, batch number, C11875500BT) supplemented with 10% fetal bovine serum (Gibco) and 1% penicillin-streptomycin (Gibco, batch number 15140-122) in 5% CO_2_ at 37°C.

MCF-7 and BT-474 cells were cultured in Opti-MEM medium in 6-well plates and transfected with lncRNA MEG3-overexpressing plasmid or a negative control vector using Lipofectamine 3000 (Invitrogen, Carlsbad, CA, USA). After 48 h, the cells were harvested and prepared for quantitative real-time PCR (qPCR) to evaluate the transfection efficiency of the lncRNA MEG3-overexpressing plasmid.

### Quantitative real-time PCR (qPCR)

Total RNA was isolated using TRIzol (Invitrogen, USA) and reversed to cDNA with the PrimeScript^™^ II 1^st^ Strand cDNA Synthesis Kit (TAKARA, Japan). The ABI Viia 7 Real-Time PCR system (Applied Biosystems, Foster City, CA) was used to perform qPCR with primer sequences listed in Table 1.

**Table 1:** The primers used in this study.

**Primer**	**Sequence (5′–3′)**
General stem-loop primer	GTGCAGGGTCCGAGGT
miR-330-5P-JH	GTCGTATCCAGTGCAGGGTCCGAG GTATTCGCACTGGATACGACGCCTAA
miR-330-5P-F	GCGTCTCTGGGCCTGTGTC
lncRNA MEG3-hF	GCTATGCTCATACTTTGACTC
lncRNA MEG3-hR	CATCATAAGGGTGATGACAG
CNN1-hF	TCTGCACATTTTAACCGAGGTC
CNN1-hR	GCCAGCTTGTTCTTTACTTCAGC
U6-RT	GTCGTATCCAGTGCAGGGTCCGAGGTATTCGCACTGGATACGACAAAATATG
U6-F	CTCGCTTCGGCAGCACA
U6-R	AACGCTTCACGAATTTGCGT
GAPDH-hF	TGACAACTTTGGTATCGTGGAAGG
GAPDH-hR	AGGCAGGGATGATGTTCTGGAGAG

### Nuclear and cytoplasmic separation PCR

Nuclear and cytoplasmic RNA fractions of MCF-7 and BT-474 cells were prepared using a PARIS kit (Thermo Fisher Scientific, USA, batch number: AM1921). Subsequently, nuclear and cytoplasmic RNA was quantified using qPCR as described above.

### Cell counting kit-8 (CCK-8) assay

Cell proliferation was examined using the CCK-8 assay. Cells cultured in a 96-well plate were added with the lncRNA MEG3-overexpressing plasmid or a negative control vector and incubated for 48 h. CCK-8 solution (10 μL) was added to terminate reactions. The optical density (OD) at 4 h was measured using a microplate reader (Bio-Rad, Hercules, CA, USA).

### Flow cytometry

Cells at 48 h post-transfection were harvested to assess cell apoptosis with Annexin V-FITC/PI Apoptosis Detection Kit (Beyotime, China). The suspended cells were stained with 5 μL Annexin V-FITC (50 μg/mL) and/or 5 μL propidium iodide (50 μg/mL) for 15 min in the dark. The cells were then analyzed using a flow cytometer (BD Biosciences, Franklin Lakes, NJ, USA).

### Transwell assay

The cell migration and invasion abilities were evaluated by Transwell inserts (Corning, NY, USA). To examine cell migration, cells in 200 μL serum-free medium were plated in the upper chamber, and 600 μL medium with 20% fetal bovine serum was added into the lower chamber. After the respective treatments, the outer membrane was fixed with 4% paraformaldehyde and stained with 0.1% crystal violet solution. The cells were observed under a microscope and photographed. For cell invasion, protocols were similar with above but the Transwell insert was mounted by Matrigel.

### Dual-luciferase reporter assay

Cells were co-transfected with luciferase reporter vector carrying wild-type or mutant lncRNA-MEG3 constructs and miR-330 mimic or mimic negative control. The luciferase activity was measured by Dual-Luciferase Reporter Assay System (Promega, Madison, WI, USA).

### Western blot

Cells were harvested to prepare cell lysates lysated by RIPA buffer. Total proteins were quantified and separated using sodium dodecyl sulfate-polyacrylamide gel electrophoresis. After transferring to a polyvinylidene fluoride membrane, it was incubated with primary antibodies including anti-CNN1 antibody (Abcam, ab46794), anti-GADPH antibody (Proteintech, 60004-1-Ig) at 4°C overnight and with goat anti-rabbit IgG (H+L)-HRP (Jackson ImmunoResearch, 111-035-003) at 23°C–26°C for 1 h. GAPDH expression was used as the loading control.

### RNA immunoprecipitation (RIP) assay

RIP experiments were conducted using the Magna RIP RNA-Binding Protein Immunoprecipitation Kit (Millipore, Billerica, MA, USA, 17-700). The antibodies used for AGO2 and control IgG were purchased from Abcam (ab186733, ab172730). The coprecipitated RNAs were detected using qPCR with total RNA as the input.

### Statistical analysis

All assays were carried out in triplicate. Quantitative data were presented as mean ± standard deviation and analyzed and visualized by GraphPad Prism 5 (GraphPad Software, San Diego, CA, USA). Statistical significance of differences between two groups or among multiple groups was analyzed using Student’s *t*-test or one-way analysis of variance (ANOVA). Statistical significance was set at *P* < 0.05.

## Supplementary Materials

Supplementary Figures

Supplementary Table 1
