# Supplementary material for: Long non-coding RNA MEG3 acts as a suppressor in breast cancer by regulating miR-330/CNN1
Source: Aging (Albany NY). 2024 Jan 17;16(2):1318–35. doi: 10.18632/aging.205419 (PMC10866439; doi:10.18632/aging.205419)
Supplement: Supplementary Figures [file aging-16-205419-s001.pdf]

SUPPLEMENTARY FIGURES

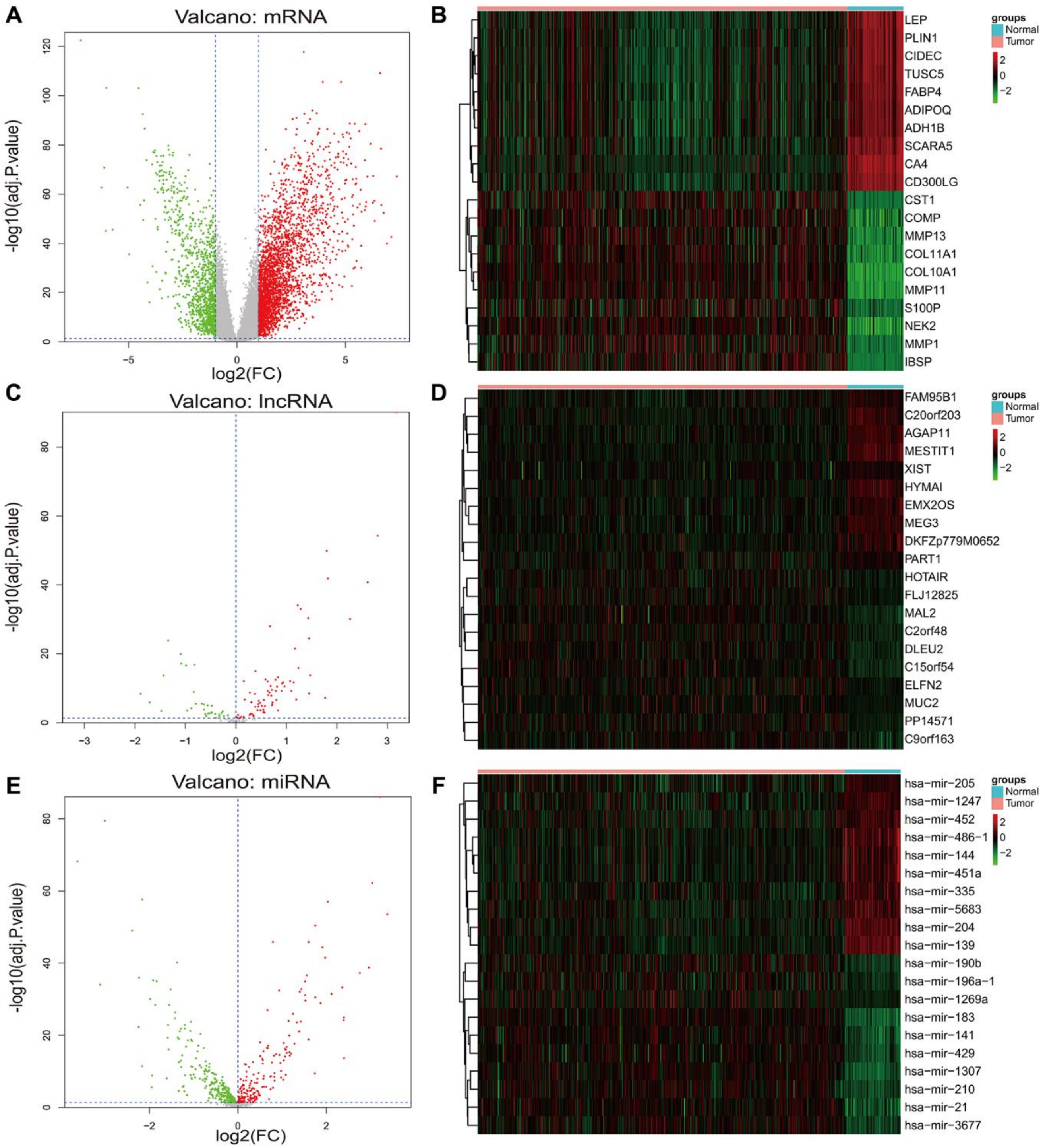

Supplementary Figure 1. Illustration of differentially expressed mRNAs, lncRNAs and miRNAs. (**A**, **B**) The volcano plot and heatmaps of differentially expressed mRNAs; (**C**, **D**) The volcano plot and heatmaps of differential lncRNAs; (**E**, **F**) The volcano plot and heatmaps of differential miRNAs. Red spots stand for up-regulated genes, while green spots stand for down-regulated genes.

A

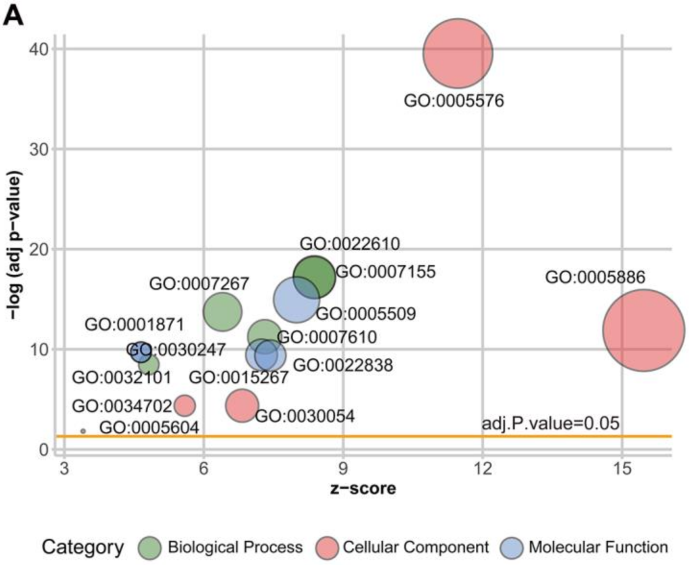

ID	Description
GO:0007155	cell adhesion
GO:0022610	biological adhesion
GO:0007267	cell−cell signaling
GO:0007610	behavior
GO:0032101	regulation of response to external stimulus
GO:0005576	extracellular region
GO:0005886	plasma membrane
GO:0030054	cell junction
GO:0034702	ion channel complex
GO:0005604	basement membrane
GO:0005509	calcium ion binding
GO:0030247	polysaccharide binding
GO:0001871	pattern binding
GO:0015267	channel activity
GO:0022838	substrate specific channel activity

B

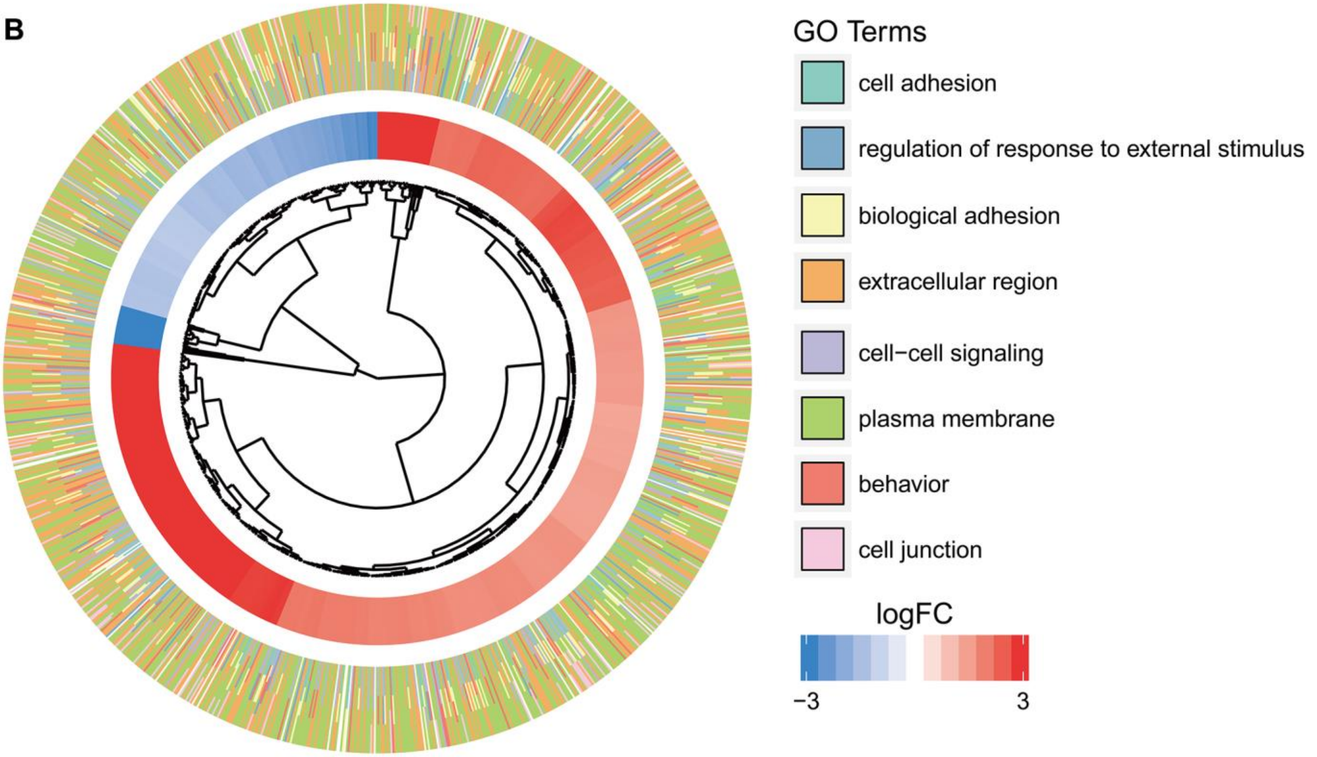

Supplementary Figure 2. Significant GO enrichment results. (**A**) The top 5 biological process (green), cellular component (red), and molecular functions (blue). (**B**) Significant GO terms and the corresponding enriched genes. Color bar from -3 to 3 represents log_2FC value.

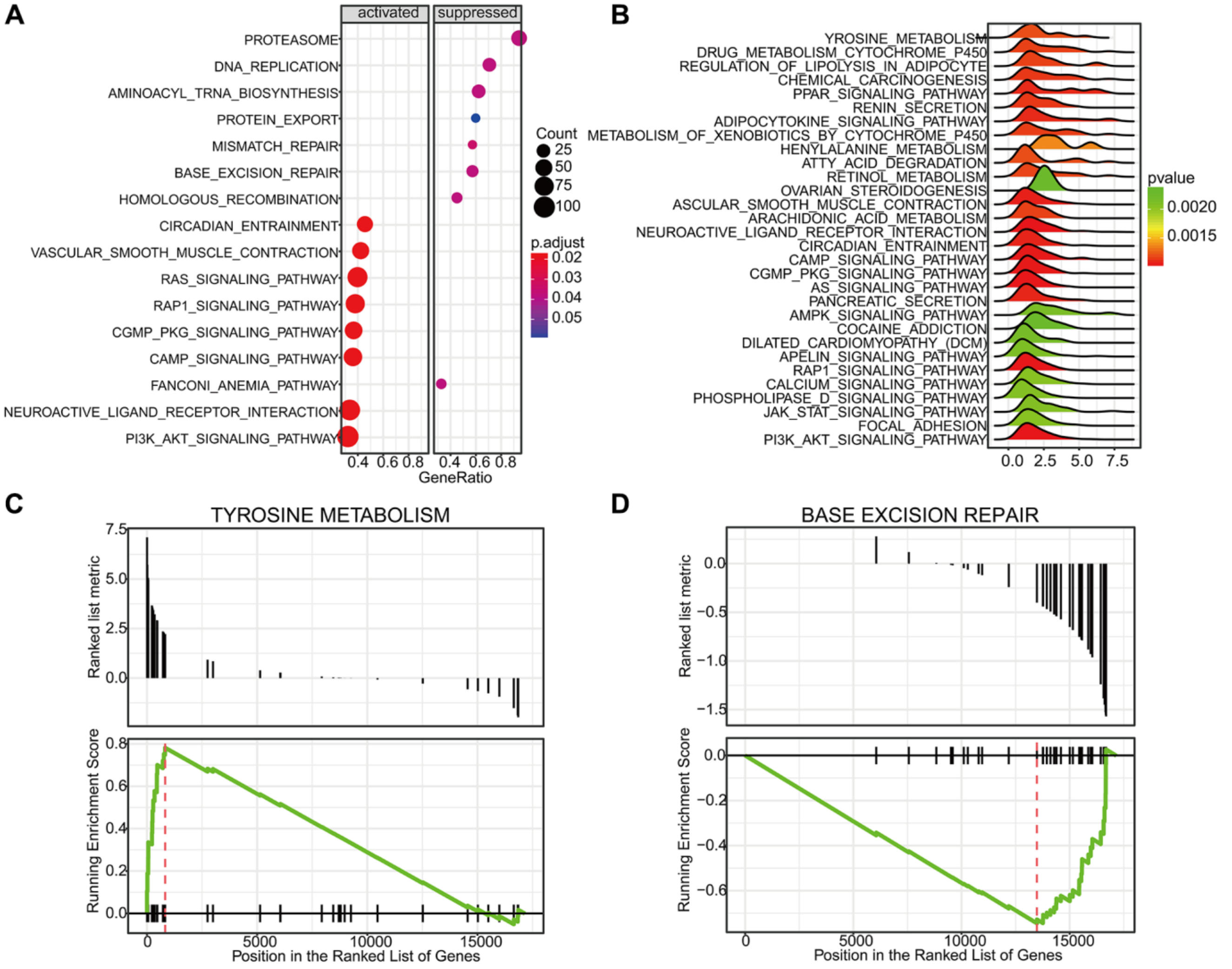

Supplementary Figure 3. GSEA enrichment pathways. (**A**) Dotplot of significant pathways. (**B**) Joyplot of significant pathways. (**C**) Up-regulated tyrosine metabolism pathway. (**D**) Down-regulated base excision repair pathway. Count, the number of genes enriched in a pathway; GeneRatio, the percentage of significantly enriched genes in all genes.
